# Novel Blind Recognition Algorithm of Frame Synchronization Words Based on Soft-Decision in Digital Communication Systems

**DOI:** 10.1371/journal.pone.0132114

**Published:** 2015-07-08

**Authors:** Jiangyi Qin, Zhiping Huang, Chunwu Liu, Shaojing Su, Jing Zhou

**Affiliations:** College of Mechatronic Engineering and Automation, National University of Defense Technology, Changsha 410073, Hunan Province, P.R. China; Jiangnan University, CHINA

## Abstract

A novel blind recognition algorithm of frame synchronization words is proposed to recognize the frame synchronization words parameters in digital communication systems. In this paper, a blind recognition method of frame synchronization words based on the hard-decision is deduced in detail. And the standards of parameter recognition are given. Comparing with the blind recognition based on the hard-decision, utilizing the soft-decision can improve the accuracy of blind recognition. Therefore, combining with the characteristics of Quadrature Phase Shift Keying (QPSK) signal, an improved blind recognition algorithm based on the soft-decision is proposed. Meanwhile, the improved algorithm can be extended to other signal modulation forms. Then, the complete blind recognition steps of the hard-decision algorithm and the soft-decision algorithm are given in detail. Finally, the simulation results show that both the hard-decision algorithm and the soft-decision algorithm can recognize the parameters of frame synchronization words blindly. What’s more, the improved algorithm can enhance the accuracy of blind recognition obviously.

## Introduction

In the digital communication system, frame synchronization is the foundation for the follow-up signal processing, such as forward error correction (FEC) and information access. Frame synchronization words can mark the place for mapping receiving binary stream and eliminating redundant information. However, the frame synchronization words parameters are different across the various communication systems. In background of the non-cooperation communication system, the blind recognition of frame synchronization words is necessary for obtaining the information contained in the unknown binary signal [1–4]. In this paper, a novel blind recognition algorithm of frame synchronization words is proposed. The algorithm exploits the soft-decision to enhance the accuracy of blind recognition. Comparing with conventional hard-decision, soft-decision utilizes the confidence level of each receiving data to recognize parameters [5–9]. The QPSK signal is used to verify the blind recognition algorithm and the simulations results show that soft-decision is better than the hard-decision.

The rest of paper is organized as follow. In section 2, the basic frame synchronization words blind recognition method is described in detail. In section 3, the improved blind recognition algorithm of frame synchronization words is shown. In section 4, simulations result is shown to verify the algorithms performance. Finally, section 5 concludes this paper.

## Materials and Methods

**Fig 1:**
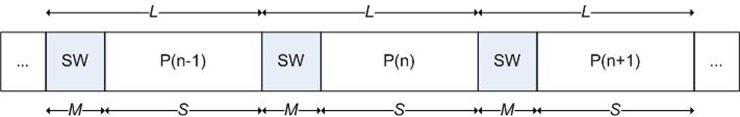
Conventional frame form.

Signal is transmitted in the form of frames. The frame alignment is used to realize the synchronization on the receiver and transmitter. The conventional frame form is shown in Fig 1. Each frame has two parts, one is synchronization words (SW), and the other is payload (P). On the transmitter, the payload is divided into groups. The length of each group is *L* and the length of synchronization words is *M*. A payload group and a synchronization words constitute a frame. On the receiver, the synchronization words are periodic emergence. However, the payload data is different between each frame. Therefore, the receiver can detect the place of synchronization words in the data stream to find the start point of each frame. Then, the receiver can obtain payload data from the data stream for other signal processing.

### Frame synchronization words blind recognition

In background of the non-cooperation communication, the frame length (*L*), synchronization words form and synchronization words length (*M*) are unknown. In order to obtain the payload data for other signal processing, these parameters should be recognized firstly [10]. According to frame synchronization words characteristic, the frame length can be recognized by detecting the periodic fragment in the data stream and confirming the period. Then, the frame synchronization words form and its length can be recognized by analyzing the periodic fragment.

**Fig 2:**
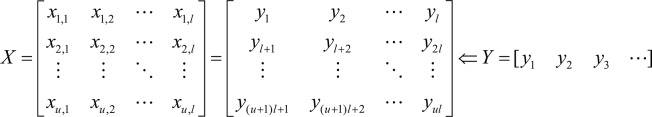
The mapping relation between matrix *X* and vector *Y*.

A *u* × *l* matrix *X* and a data stream vector *Y* are shown in Fig 2. *Y* means the receiving binary stream.

Put the data of *Y* into *X* successively. When the column number of *X* is equal to the frame length, some columns data are equal and the other columns data are random. The former are synchronization columns and the latter are non-synchronization columns. When the column number of *X* is not equal to the frame length, each columns data are random. The synchronization columns will not appear. Therefore, calculate the sum of each column and use the sum to represent the number of “1”. If the number of “1” in a column is closed to 0 or *u*, this column can be recognized as the frame synchronization words column. Based on above methods, traverse the *L* value. Then, the frame length and frame synchronization words can be recognized. In order to distinguish the non-synchronization columns and synchronization columns, reasonable thresholds should be designed [11–12]. And the column number of *X* is important to improve fault-tolerant ability and reduce calculation.

The columns can be expressed as follow.

$$X_{j}={\left(x_{1,j},x_{2,j},\ldots ,x_{u,j},\right)}^{T}\left(1\leq j\leq l\right)$$ (1)

When the column is non-synchronization column, each data in it is random. Therefore, the probability of “0” and the probability of “1” are equal to 0.5 approximately.

*Z*_*j*_ is the sum of the *j* column. Therefore, *Z*_*j*_ obeys the binomial distribution and its mathematical expectation and variance can be expressed as follow.

$$\left\{ \begin{array}{l} E\left(Z_{j}\right)=u\times {\text{P}}_{r}\left(x_{i,j}=1\right)=u/2\\ D\left(Z_{j}\right)=u\times {\text{P}}_{r}\left(x_{i,j}=1\right)\times {\text{P}}_{r}\left(x_{i,j}=0\right)=u/4 \end{array}\right.$$ (2)

When it is synchronization column and there is no bit error in the column, every data in the column is “0”, or everyone is “1”. Suppose the channel conversion probability is *τ* and channel noise is existed. The bit flipping probability can be expressed as follow.

$$\left\{ \begin{array}{l} {\text{P}}_{r}\left(x_{i,j}=\text{s}\right)=1-\tau \\ {\text{P}}_{r}\left(x_{i,j}=1-\text{s}\right)=\tau \end{array}\right.,\left(1\leq i\leq u,1\leq j\leq l\right)$$ (3)

Where *s* (*s* = 1 or 0) is transmission bit.

In this case, *Z* ^*^_j_ means the sum of *j* column. *Z* ^*^_j_ obeys the binomial distribution and its mathematical expectation and variance can be expressed as follow.

$$\left\{ \begin{array}{l} E\left(Z^{*}{}_{j}\right)=u\times {\text{P}}_{r}\left(x_{i,j}=1\right)=u(\tau -2s\tau +s)\\ D\left(Z^{*}{}_{j}\right)=u\times {\text{P}}_{r}\left(x_{i,j}=1\right)\times {\text{P}}_{r}\left(x_{i,j}=0\right)=u\tau (1-\tau ) \end{array}\right.$$ (4)

In order to balance the false alarm probability and false dismissal probability, according to the six times standard deviation principle, the average of non-synchronization and synchronization columns six times standard deviation boundary value is used as the decision threshold. The decision threshold decides which column is synchronization column and which is not. And, there are two kinds of synchronization column. One is made up of “0” and the other is made up of “1”. Therefore, there should be two kinds of threshold, and they can be expressed as follow.

$$\left\{ \begin{array}{l} \delta_{up}=\frac{\left(\frac{u}{2}+6\times \sqrt{\frac{u}{4}}\right)+\left[u\left(1-\tau \right)-6\times \sqrt{u\tau \left(1-\tau \right)}\right]}{2}\\ \delta_{down}=\frac{\left(\frac{u}{2}-6\times \sqrt{\frac{u}{4}}\right)+\left[u\tau -6\times \sqrt{u\tau \left(1-\tau \right)}\right]}{2} \end{array}\right.$$ (5)

When the value of *Z* ^*^_j_ is between the *δ*_*up*_ and *δ*_*down*_, the *j* column is decided to be non-synchronization column. Otherwise, it would be decided to be synchronization column. According to (5), another relation can be deduced as follow.

$$\left\{ \begin{array}{l} \left(\frac{u}{2}+6\times \sqrt{\frac{u}{4}}\right)<\left[u\left(1-\tau \right)-6\times \sqrt{u\tau \left(1-\tau \right)}\right]\\ \left(\frac{u}{2}-6\times \sqrt{\frac{u}{4}}\right)<\left[u\tau -6\times \sqrt{u\tau \left(1-\tau \right)}\right] \end{array}\right.$$ (6)

Therefore, the *u* should be confirmed with the relation as follow.

$$u>\frac{36\left[1+2\sqrt{\tau \left(1-\tau \right)}\right]}{{\left(1-2\tau \right)}^{2}}$$ (7)

However, the *τ* is unknown in the blind recognition and the *Z* ^*^_j_ probability distribution is not dependent on *τ*. Therefore, *δ*_*up*_ and *δ*_*down*_ can be expressed as follow.

$$\left\{ \begin{array}{l} \delta_{up}=\left(\frac{u}{2}+6\times \sqrt{\frac{u}{4}}\right)=\frac{1}{2}(u+6\sqrt{u})\\ \delta_{down}=\left(\frac{u}{2}-6\times \sqrt{\frac{u}{4}}\right)=\frac{1}{2}(u-6\sqrt{u}) \end{array}\right.$$ (8)

In order to ensure the validity of decision, the difference of *δ*_*up*_ and *δ*_*down*_ should be small.

$$\delta_{up}-\delta_{down}<u\theta (0<\theta <1)$$ (9)

Then, according to (8), *u* can be expressed as follow.

u>36θ2 (10)

When *θ* is equal to 0.5, the *u* should be larger than 144.

### The improve blind recognition algorithm

**Fig 3:**
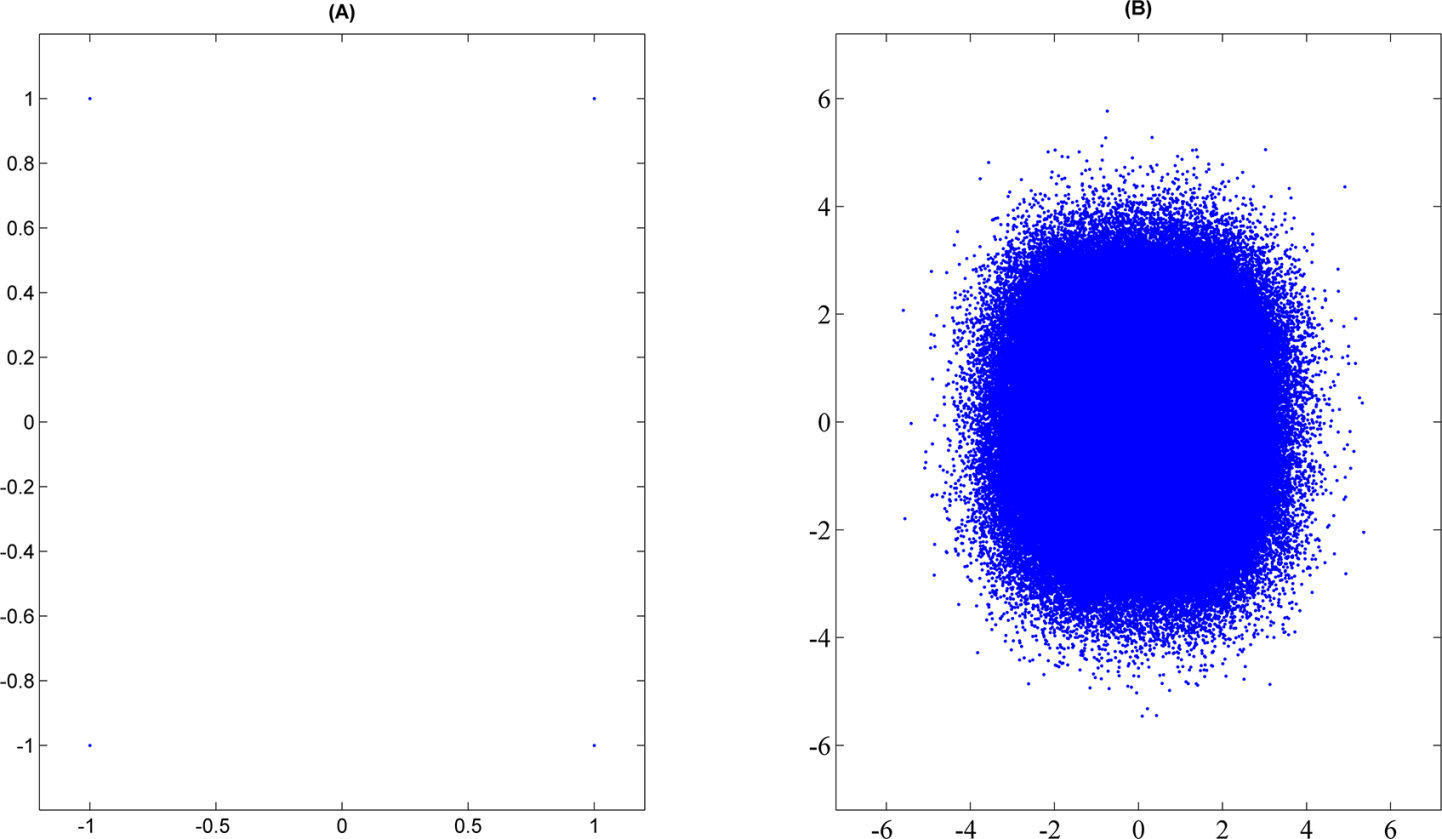
(A) The constellation diagrams at the transmitter (B) The constellation diagrams at the receiver.

In section 2, a basic frame synchronization words blind recognition method is proposed. The method is based on the hard-decision. In this section, an improved blind recognition algorithm based on the soft-decision will be proposed. QPSK signal will be used to describe the improved algorithm. Suppose the QPSK signal transmitted through the Additive White Gaussian Noise (AWGN) channel and the transmitted signal is random. The QPSK signal constellation diagrams on the transmitter and the receiver are shown in Fig 3. There are four types of transmission signals on the transmitter. They are **s**_1_ = 1 + *i*, **s**_2_ = −1 + *i*, **s**_3_ = −1 − *i* and **s**_4_ = 1 − *i*. The transmission signals in AWGN channel have been distorted. Therefore, these signals cannot be used directly on the receiver. The constellation diagrams on the transmitter and receiver are shown in Fig 3.

The data near the real axis and the imaginary axis will lead to misjudgment. Therefore, in each column of *X*, a certain number of unreliable receiving data should be deleted. The confidence level of each receiving data can be expressed as follow.

$$g_{j}=\underset{i=1,2,3,4}{\text{max}}\left\{\left|r_{j}-s_{i}\right|\right\}$$ (11)

The data with a greater degree of confidence level will be reserved and the data with a lower degree of confidence level may be deleted.

The complete steps of the improved blind recognition algorithm are shown as follow.

1. Set the frame length searching scope (*l*_min_ and *l*_max_), initialize *l* as *l*_min_. Calculate *u*, *δ*_*up*_ and *δ*_*down*_.
2. Put the receiving data into the *u* × *l* matrix *X*.
3. Calculate the confidence level of each receiving data. The receiving data in the bottom fifth of confidence level will be deleted. The reserved data constitute the u^'^ × *l* matrix *X* ^'^. The *u* ^'^ is equal to ⌊0.8×*u*⌋.
4. Calculate the sum of each column in the, put the result into the row vector *U*_*l*_ and the vector length is *l*.
5. Search elements which are greater than *δ*_*up*_ or less than *δ*_*down*_ in *U*_*l*_. Record the sum of these elements as *Q*_*l*_, calculate the *V*_*l*_ = *Q*_*l*_ / *l*.
6. If *l* < *l*_max_, *l* = *l* + 1 and go to (2), otherwise, go to (7).
7. The vector *V* can be expressed as $\left[V_{l}{}_{{}_{\text{min}}},V_{l}{}_{{}_{\text{min}}+1},\cdots ,V_{{{}_{l}}_{{}_{\text{max}}}}\right]$, record the maximum value of *V* as *V*_max_.
8. Search the elements which are larger than (2 / 3)×*V*_max_, and the minimum subscript of these elements will be the estimation of frame length, record as $\hat{L}$.
9. Make *l* equal to $\hat{L}$, put the reserved data into *X* ^'^ again and calculate the sum of each column in the matrix, put the result into $U_{\hat{L}}$.
10. Select the data which are greater than *δ*_*up*_ or less than *δ*_*down*_ from $U_{\hat{L}}$. The data with consecutive subscript will be the recognition of frame synchronization words. If the data is greater than *δ*_*up*_, the corresponding position bit of frame synchronization words is "1". Otherwise, it is "0". And the number of consecutive subscript is the length of frame synchronization words.

## Results and Discussion

In this section, simulations are used to verify the validity of the frame synchronization words blind recognition algorithms proposed in section 2 and section 3. Then a comparison will be made to test the performance between the algorithm based on hard-decision and the one based on soft-decision.

The simulations use the QPSK signal communication system and transmission channel is AWGN channel. Meanwhile, the assumptions proposed in section 2 and section 3 are true. In the simulations, the length of frame is 1024 signs, the frame synchronization words is 0xF628 and the *u* is 150. The value of *l*_min_ is 970 signs and the value of *l*_max_ is 1070 signs. The Signal Noise Ratio (SNR) is 3dB, and the constellation diagrams on the transmitter and on the receiver are shown in Fig 3. To simplify calculation, the normalizing parameter *Z* ^'^_*j*_, *δ* ^'^_*up*_ and *δ* ^'^_*dowm*_ are used to replace the *Z*_*j*_, *δ*_*up*_ and *δ*_*down*_.

**Fig 4:**
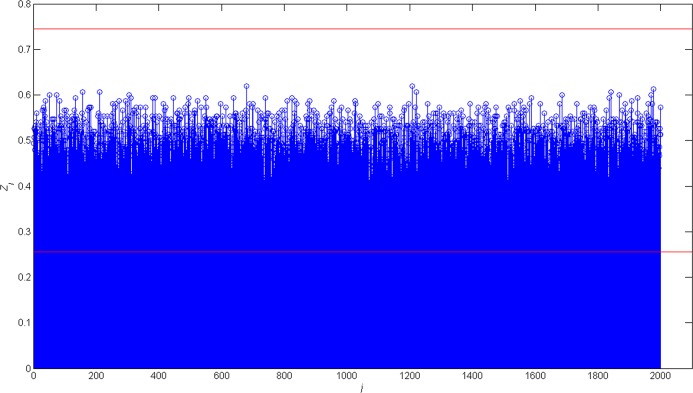
The value of *Z* ^'^ when *l* ≠ *L*.

**Fig 5:**
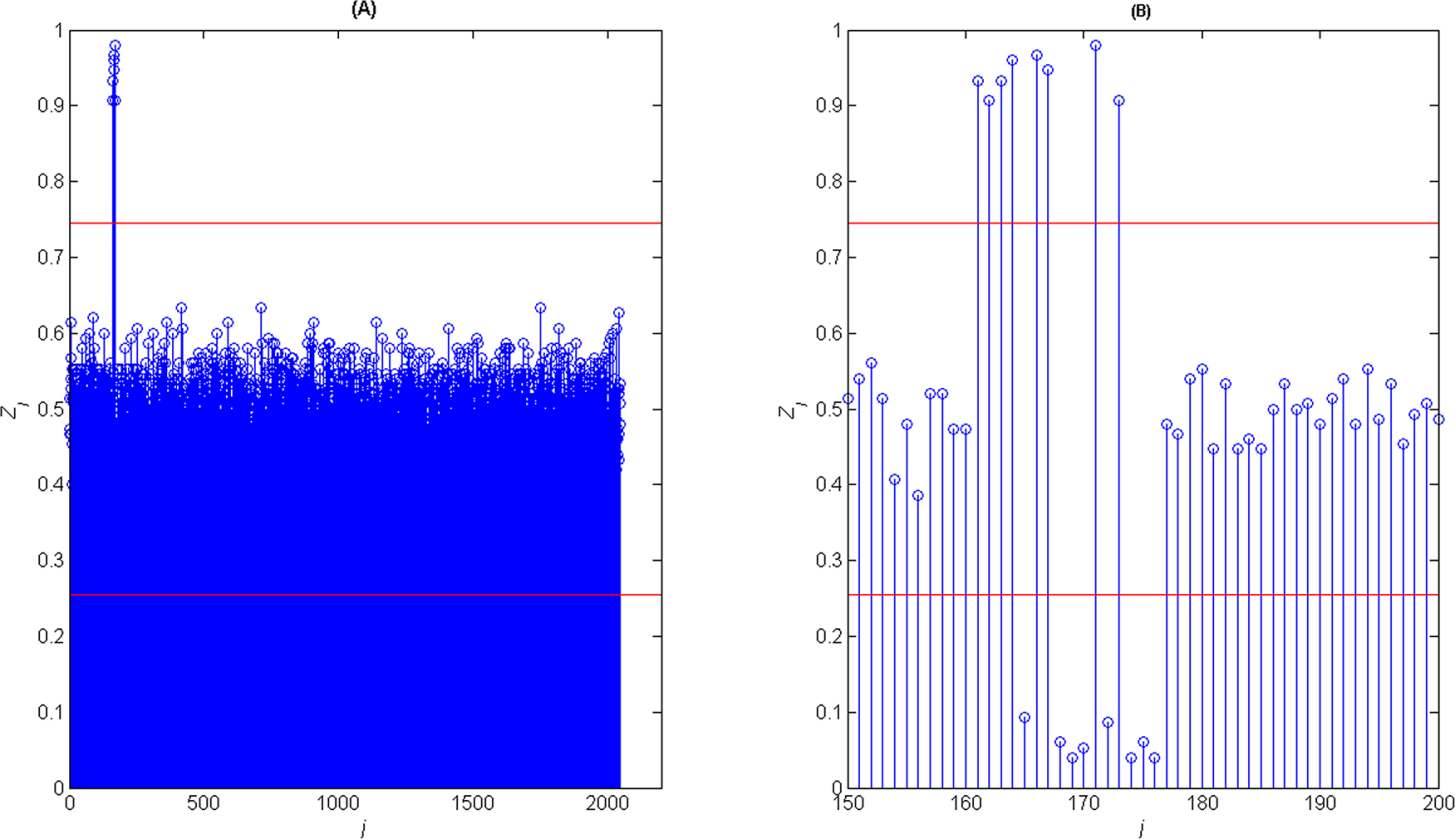
(A) The value of *Z* ^'^ (B) The partial zoom of *Z* ^'^.

According to the steps proposed in section 3, the simulation results of blind recognition algorithms based on soft-decision are shown in Fig 4 and Fig 5. The red lines in Fig 4 and Fig 5 mean the *δ* ^'^_*up*_ and the *δ* ^'^_*dowm*_. The value of *δ* ^'^_*up*_ and the *δ* ^'^_*dowm*_ can be calculated according to (8).

When the *l* = 1023, there isn't any *Z* ^'^_*j*_ greater than *δ* ^'^_*up*_ or smaller than *δ* ^'^_*down*_ in Fig 4. Therefore, the frame synchronization words cannot be recognized.

When the *l* = 1024, there are several *Z* ^'^_*j*_ greater than *δ* ^'^_*up*_ or smaller than *δ* ^'^_*down*_ in Fig 5. These columns can be used to recognize the frame synchronization words. The partial zoom figure is shown in Fig 5. From the partial zoom figure, the frame synchronization words can be recognized easily.

**Fig 6:**
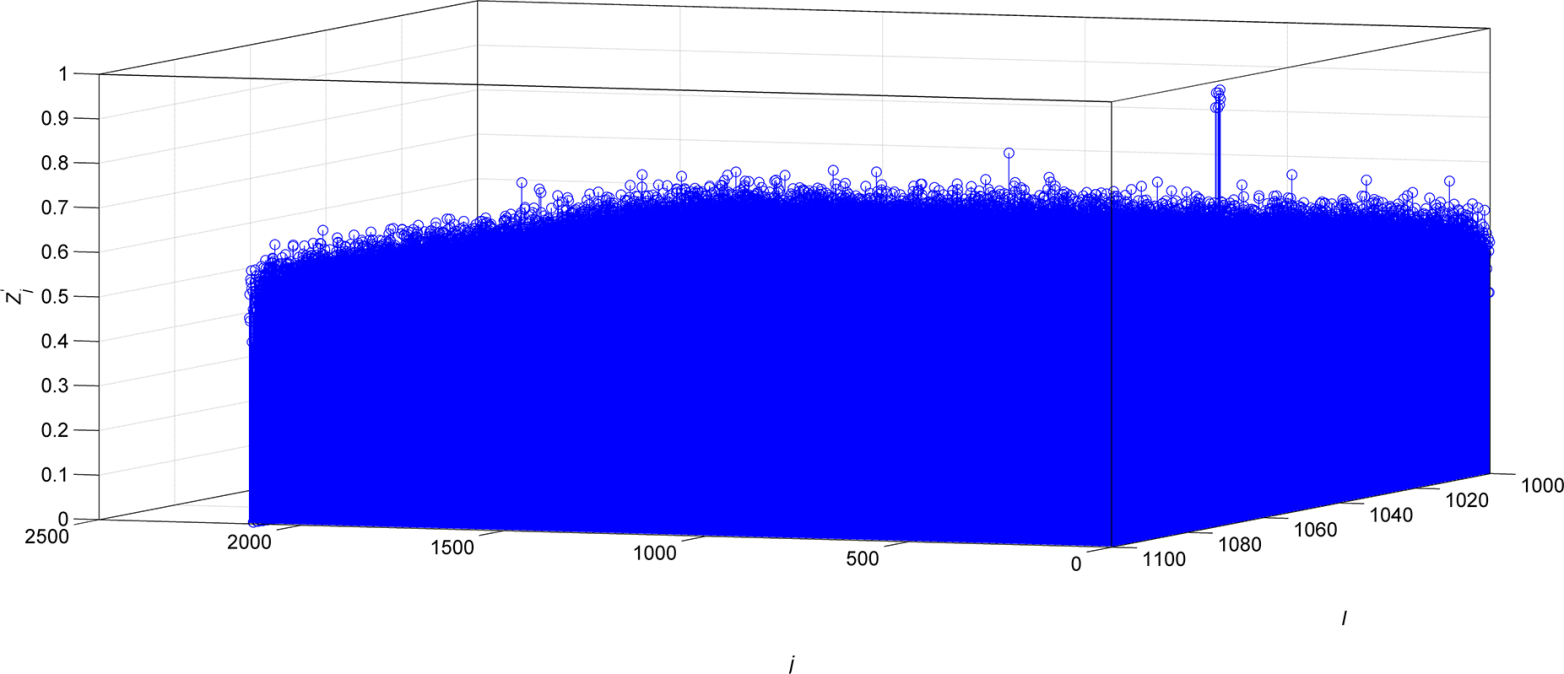
The result of frame length recognition.

To recognize the frame length, *l* should be traversed from the *l*_min_ to *l*_max_, the search results are shown in Fig 6.

In Fig 6, only when *l* is equal to 1024, there are several the *Z* ^'^_*j*_ meet the requirement. Therefore, according to the simulation results, the frame synchronization words are recognized as 0xF628 and the recognition result is correct.

**Fig 7:**
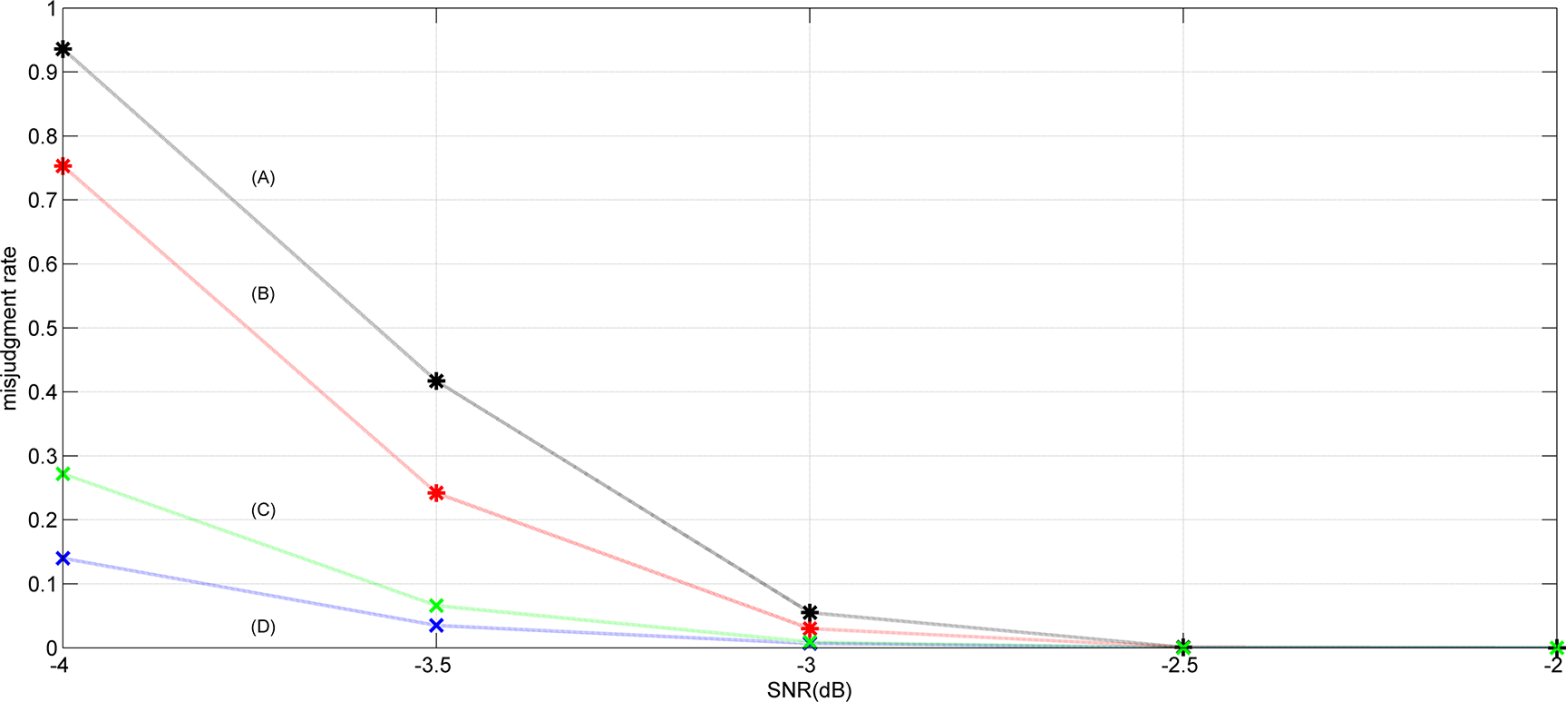
(A) Hard-decision result of 32 bits frame synchronization words (B) Soft-decision result of 32 bits frame synchronization words (C) Hard-decision result of 16 bits frame synchronization words (D) Soft-decision result of 16 bits frame synchronization words.

To compare the performance between the hard-decision and the soft-decision, the compare simulation results are shown in Fig 7. In the simulations, different AWGN is adding to the channel and recognize the frame synchronization words with different lengths. The 0xF628 and 0xF628F628 are used respectively as the frame synchronization words in these simulations. Through 1000 simulations, the misjudgment rates of blind recognition are shown in Fig 7. The simulations curves mean that the soft-decision results are better than the hard-decision results. And the length of frame synchronization words will impact the misjudgment rate.

## Conclusions

In this paper, a novel blind recognition algorithm of frame synchronization words is proposed. Comparing with the algorithm based on hard-decision, the one based on soft-decision is developed to improve the recognition performance. QPSK signal is used to test the performance of these algorithms. The simulation results mean that these algorithms can recognize frame synchronization words and soft-decision is better than hard-decision. What is more, these algorithms can be used in other signal modulation forms.
